# Functional Brain Connections Identify Sensorineural Hearing Loss and Predict the Outcome of Cochlear Implantation

**DOI:** 10.3389/fncom.2022.825160

**Published:** 2022-03-30

**Authors:** Qiyuan Song, Shouliang Qi, Chaoyang Jin, Lei Yang, Wei Qian, Yi Yin, Houyu Zhao, Hui Yu

**Affiliations:** ^1^College of Medicine and Biological Information Engineering, Northeastern University, Shenyang, China; ^2^Key Laboratory of Intelligent Computing in Medical Image, Ministry of Education, Northeastern University, Shenyang, China; ^3^Department of Electrical and Computer Engineering, University of Texas at El Paso, El Paso, TX, United States; ^4^Department of Radiology, The Affiliated Hospital of Guizhou Medical University, Guiyang, China; ^5^Department of Otolaryngology, The Affiliated Hospital of Guizhou Medical University, Guiyang, China; ^6^Department of Radiology, The Seventh Affiliated Hospital, Southern Medical University, Foshan, China

**Keywords:** sensorineural hearing loss, resting-state fMRI, functional brain network, cochlear implantation, machine learning, multiple logistic regression

## Abstract

Identification of congenital sensorineural hearing loss (SNHL) and early intervention, especially by cochlear implantation (CI), are crucial for restoring hearing in patients. However, high accuracy diagnostics of SNHL and prognostic prediction of CI are lacking to date. To diagnose SNHL and predict the outcome of CI, we propose a method combining functional connections (FCs) measured by functional magnetic resonance imaging (fMRI) and machine learning. A total of 68 children with SNHL and 34 healthy controls (HC) of matched age and gender were recruited to construct classification models for SNHL and HC. A total of 52 children with SNHL that underwent CI were selected to establish a predictive model of the outcome measured by the category of auditory performance (CAP), and their resting-state fMRI images were acquired. After the dimensional reduction of FCs by kernel principal component analysis, three machine learning methods including the support vector machine, logistic regression, and k-nearest neighbor and their voting were used as the classifiers. A multiple logistic regression method was performed to predict the CAP of CI. The classification model of voting achieves an area under the curve of 0.84, which is higher than that of three single classifiers. The multiple logistic regression model predicts CAP after CI in SNHL with an average accuracy of 82.7%. These models may improve the identification of SNHL through fMRI images and prognosis prediction of CI in SNHL.

## Introduction

Congenital sensorineural hearing loss (SNHL) occurs in 0.2–0.4% of live births. It affects approximately 40,000 children in the United States (US) each year, and nearly two-thirds of cases result in bilateral hearing loss (Prosser et al., 2015; Lieu et al., 2020). In China, approximately 30 million people suffer from congenital SNHL and there are ∼23,000 newborn deaf children and ∼50–60,000 late-onset deafness patients every year (National Bureau of Statistics of the People’s Republic of China [NBSPRC], 2007). Hearing loss results from the blockage or attenuation of auditory input to the brain and changes the connectivity and processing of the auditory stimulus by the brain (Halliday et al., 2017; Wilson et al., 2017). Bilateral SNHL can impair the speech development of children and even result in difficulties in socialization and poor academic performance (Tuller and Delage, 2014). Among numerous treatments, cochlear implantation (CI) is highly effective for SNHL (Kral and O’Donoghue, 2010). Therefore, early identification, determination of the cause and appropriate treatment plans are essential for the patient’s recovery.

Medical imaging assumes a crucial role in the diagnostic evaluation of congenital SNHL. In addition to determining the underlying cause, it can also identify related abnormalities caused by hearing loss and evaluate the applicability of surgical intervention (Woolley et al., 1997; Jackson et al., 2015; Gillard et al., 2020). Preferred imaging modalities for evaluating children’s SNHL are high-resolution computed tomography (HRCT) and magnetic resonance imaging (MRI) of the temporal bone. The diagnosis rate of HRCT is ∼30% (Chen et al., 2014). MRI can detect more brain abnormalities and has a higher diagnosis rate than HRCT (Buchman et al., 2006; Ratnanather, 2020). In particular, MRI is highly effective in identifying cochlear nerve defects, which are common in SNHL (Manno et al., 2021).

Previous studies reported significant differences in the MRI-based cerebral volume and gray matter microstructure in children with SNHL (Moon et al., 2020). Hearing loss may affect white matter tracts linking the eighth cranial nerve to subcortical nuclei (i.e., cochlear) and primary auditory cortices (i.e., Heschl’s gyri) and the gray matter of primary auditory cortices (Feng et al., 2018; Tarabichi et al., 2018). Cross-modal reorganization in the auditory deprived cortex was extensively reported (Lomber et al., 2010; Ding et al., 2015). Specifically, for sensory inputs such as hearing, vision, and touch in individuals who lack one sensory mode, another sensory mode can “take over” the cortical area belonging to the lacking sensory mode (Neville et al., 1998; Sadato et al., 2004). Studies involving animal autopsies support this cross-modal plasticity. Cell structure changes were observed in the auditory cortex of deaf cats, whose magnitudes are related to the age of onset of deafness (Butler and Lomber, 2013).

Functional magnetic resonance imaging (fMRI) is a non-invasive technique that can be used to study brain function changes in a variety of diseases, providing valuable information for explaining pathogenesis and guiding clinical practice (Mulders et al., 2015; Puschmann and Thiel, 2017; Vos et al., 2017; Xia et al., 2017; Tang et al., 2019; Jin et al., 2021). According to previous studies, resting-state fMRI (rs-fMRI) can reflect underlying neuronal activity (Logothetis, 2002; Qi et al., 2015; Zhu et al., 2019; Qian et al., 2021). The analysis of functional connections (FCs) calculates the temporal correlation of blood-oxygen-level-dependent (BOLD) signal fluctuations between brain regions. A positive FC indicates that the activity between two voxels or brain regions is synchronized. Recent studies with FC show that SNHL infants exhibit functional reorganization of the auditory network in the early stage of the sensitive or critical period (Wang et al., 2019, 2021; Chen et al., 2020; Cui et al., 2022). Alterations in the regional homogeneity measured by rs-fMRI have also been reported in the auditory, visual, motor, and other related brain cortices for children with congenital SNHL (Guo et al., 2021).

Accurate prediction of the clinical outcome after CI will help project realistic expectations of the benefit for each patient with SNHL, prepare additional rehabilitation for patients with under-performance, and even improve the implantation criteria and procedures (Velde et al., 2021). The CI outcome is assessed by the categories of auditory performance (CAP) score, which can be predicted by the preoperative auditory brainstem response (ABR) and the area ratio of the vestibulocochlear nerve to the facial nerve (Han et al., 2019). Using demographic, audiological and hearing-related clinical history, as well as etiology features, machine learning models can outperform linear ones in the prediction of CI outcomes in adult patients, although their overall accuracy remains limited (Shafieibavani et al., 2021).

It is noted that by using rs-fMRI images, the accurate identification method of SNHL and good prognostic prediction of CI are not well investigated. In this study, we propose to combine FCs measured by rs-fMRI and machine learning to identify SNHL and predict the outcome of CI measured by CAP. To the best of our knowledge, this is the first such reported study.

## Materials and Methods

### Participants

**FIGURE 1:**
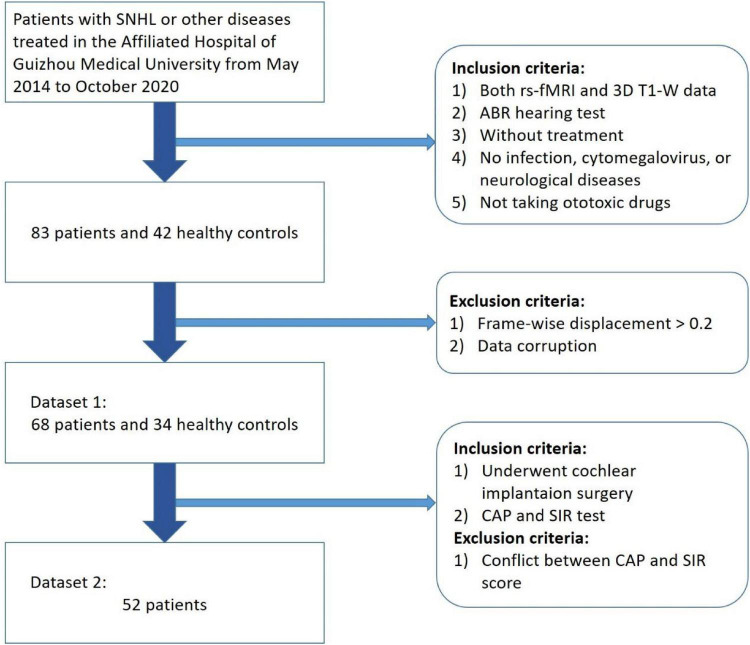
Participant selection procedure (a total of 68 children patients with congenital SNHL and 34 health controls were selected as Dataset 1 and 52 patients who underwent cochlear implantation were selected as Dataset 2).

Initially 83 children with congenital SNHL aged 0–11 years prior to undergoing CI surgery from May 2014 to October 2020 at the Affiliated Hospital of Guizhou Medical University were selected in this study (Figure 1). At the same time, we selected 42 patients for the normal hearing control group, who attended the hospital for other treatments.

All children were subjected to hearing screening. Patients with an auditory brainstem response (ABR) higher than 90 dB were considered to have severe bilateral hearing loss. All deaf children participating in the study had not worn hearing aids, had not taken ototoxic drugs, nor had a history of cytomegalovirus, infection, trauma, or any other neurological disease. Due to the relatively young age of the subjects in this study, patients were orally administered 10% chloral hydrate solution with a dosage of 0.5 mL/kg to ensure the quality of the scan. Before the examination, all of the subjects’ parents signed an informed consent form. This study was approved by the ethics committee of the Affiliated Hospital of Guizhou Medical University.

During image preprocessing, 15 SNHL and 8 HC children were excluded due to head movement and data corruption such as missing files, errors in file format conversion, etc. The remaining 68 SNHL patients and 34 HCs constituted Dataset 1. Among the 68 SNHL patients, 53 patients who underwent CI surgery took the CAP test and the speech intelligibility rating (SIR) test. One CI device was implanted into the unilateral ear with the lower hearing loss measured by ABR for each patient. All CI devices were provided by the same brand and manufacturer. No related complications were observed in the children involved in this study. One patient was excluded due to a conflict between CAP and SIR scores (CAP = 1; SIR = 3). Finally, 52 SNHL patients who underwent CI constituted Dataset 2.

### Magnetic Resonance Imaging Image Acquisition

The MR images of all participants were acquired before receiving any treatment. In this study, we used a Philips Achieva 3.0TX series MR scanner with eight-channel phased coils. The high-resolution T1-weighted images were obtained with the following parameters: echo time (TE) = 4.6 ms, repetition time (TR) = 9.4 ms, flip angle (FA) = 8°, slice thickness = 1.6 mm, slice interval = 0.8 mm, field of view (FOV) = 220 × 220 mm^2^, acquisition matrix = 276 × 227 (i.e., the pixel spacing was 0.797 and 0.969 mm, respectively).

Using the echo plane imaging sequence, the BOLD-based functional MR images were obtained with the flowing the parameters: TE = 30 mm, TR = 2000 ms, time point = 200, FA = 90°, FOV = 220 × 220 mm^2^, slice thickness = 3.40 mm, the number of slices = 35.

### Study Procedure

**FIGURE 2:**
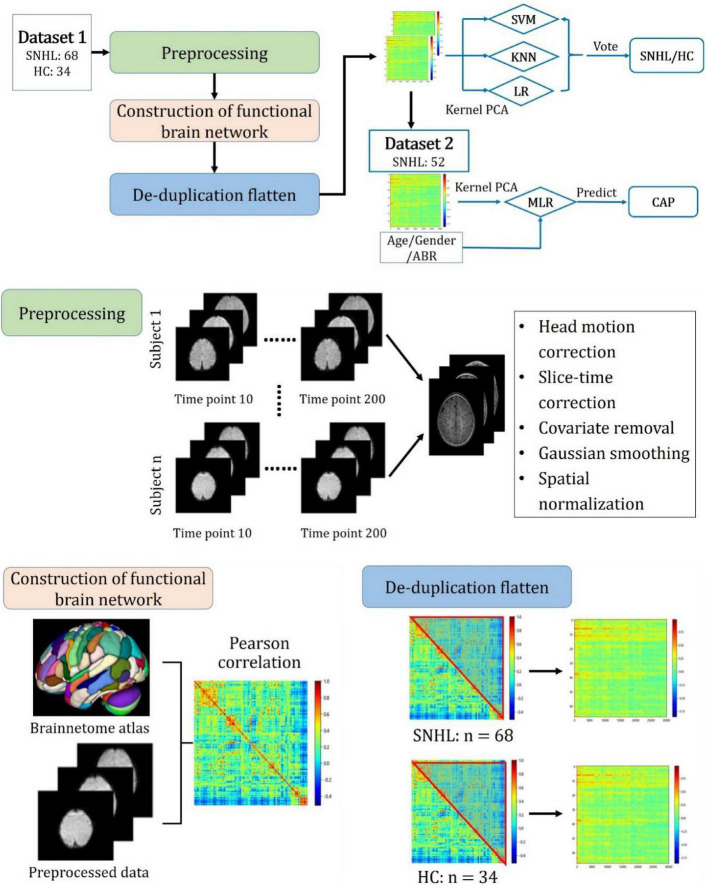
Overall study procedure with main steps including preprocessing, construction of functional brain network, de-duplication flattening, construction of machine learning models for SNHL identification, and building of multiple logistic regression model for predicting the outcome after cochlear implantation measured by the category of auditory performance.

The overall study procedure is illustrated in Figure 2. It comprises two main tasks, namely, (1) classification of SNHL and HC and (2) prediction of CAP after CI in SNHL. For task (1), Dataset 1 was used with five main steps: (I) Image preprocessing; (II) Construction of the functional brain network; (III) De-duplication and flattening; (IV) Dimensionality reduction by kernel principal component analysis (KPCA); (V) Classification by machine learning methods. Task (2) employed Dataset 2. After the dimensionality reduction, the obtained principal components and parameters of age, gender, and ABR were used to build a multiple logistic regression (MLR) model to predict CAP after CI in SNHL. The details of each task and step are given as follows.

### Data Preprocessing and Construction of Brain Networks

We preprocessed rs-fMRI images using the Matlab R2018a platform with data processing and analysis for brain imaging (DPABI) and statistical parameter mapping (SPM) software SPM12. First, we converted the files from the DICOM format into a standard NIFTI format. Second, the first 10 time-points in the serial of rs-fMRI data were discarded to avoid errors caused by unstable magnetic fields. Third, the slice time correction, head motion correction, and covariate removal were performed on rs-fMRI data. Fourth, the initial coordinates of T1-weighted images (T1WI) and fMRI images were manually located to the anterior commissure, and images with excessive displacement and rotation deviations were corrected manually. Fifth, the processed images were normalized to the space of the Montreal Neurological Institute (MNI). Finally, the images were filtered and smoothed using a Gaussian filter with the default setting. Instances where the average frame displacement of the image exceeded 0.2 were considered to have exceeded the allowed head movement were thus removed from the data set.

Preprocessed rs-fMRI data were used to construct the functional brain network. In this study, the Human Brainnetome Atlas released by the Chinese Academy of Sciences was applied to parcellate the whole brain into 246 subregions (Fan et al., 2016). The Pearson coefficient between time series in different subregions can be calculated, which is referred to as the FC. After eliminating the double-counted FCs, we finally obtained a vector of 30,135 FCs for each participant.

### Dimension Reduction Method

The principal component analysis (PCA) is a dimension reduction method that transforms multiple related original variable indicators into several independent comprehensive ones. It uses several important principal components to explain most original data and improve analysis efficiency. In the case of a non-linear attribute in the original data, the principal components extracted by principal component analysis cannot reflect this non-linear attribute. Kernel PCA (KPCA) is a non-linear transformation based on the original data, which extracts the non-linear relationship between the data. Therefore, KPCA is adopted in this study.

Moreover, the selection of optimal kernel functions and the number of features for different classifiers was conducted by the grid search method. The Kernel function can choose a linear, sigmoid, poly, radial basis function (RBF) kernel. Studies have shown (Gillies et al., 2016) that the feature number is generally selected as 10% of the sample size to obtain a better classification effect. Hence, this study selected 10 features as the benchmark, and the number of features is searched from 2 to 20.

### Machine Learning Model

We selected three Models of support vector machine (SVM), logistic regression classifier (LR) and nearest neighbor algorithm classifier (KNN) as machine learning classifiers. The voting was conducted by prediction of the three classifiers. The voting method involves averaging the predicted probabilities of the samples according to different classifiers. To ensure the best performance of the classifier, the internal hyperParameters of the machine learning classifier and the parameters of the KPCA are simultaneously determined by the grid search. We performed 10-fold cross-validation on Dataset 1 and subsequently combined the results of each fold to obtain the final precision, recall, F1-score, accuracy, and area under curve (AUC).

### Prediction of Category of Auditory Performance After Cochlear Implantation

Because the CAP test results are all integer ratings, this study employs a multiple logistic regression method to perform a multi-class analysis on Dataset 2. Studies showed (Wu et al., 2016) that the effect of surgery is highly correlated with the age of CI. Therefore, we have introduced age as an independent variable. In Dataset 2, we preprocessed the fMRI data and calculated the FC, and then used KPCA for dimensionality reduction. Then, we selected CAP as the dependent variable; age, and gender as independent variables; principal components, and ABR data as covariates, and conducted MLR analysis using SPSS25 software.

MLR is represented as follows.


(1) $$\text{P}\left(\text{r}|x_{i}\right)=\frac{\text{exp}\,\left(w_{i}\cdot x_{i}\right)}{\sum_{j=1}^{K}\text{exp}\,\left(w_{j}\cdot x_{j}\right)}$$

Here, P denotes the posterior probability of the sample point *x_i_* belonging to the category *r*, and *x_i_* is a feature vector with dimension D. The weight vector of the i-th category is set to *w_i_*, and there is a total of K weight vectors.

## Results

### Demographic and Clinical Information

Tables 1, 2 summarize the demographic and clinical characteristics of all participants (Dataset 1) and the participants who underwent CI (Dataset 2). The age in the HC and SNHL groups was 45.62 ± 27.63 and 46.24 ± 24.38 months (mean ± SD) and the ranges 12–117 and 12–137 months, respectively. There were no significant differences in gender and age between HC and SNHL groups (*p* > 0.05).

**TABLE 1:** Demographic and clinical characteristics of all participants.

	HC group	SNHL group	*P*-value
Number	34	68	–
Age (months) (mean ± SD)*^a^*	45.62 ± 27.63	46.24 ± 24.38	0.908
Age range (months)	12–117	12–137	–
Gender (male/female)*^b^*	18/16	35/33	0.889

*HC, health control; SD, standard deviation; SNHL, sensorineural hearing loss.*

*^a^Statistical analysis is done by two-sample t-test.*

*^b^Statistical analysis is done by Chi-square test.*

**TABLE 2:** Demographic and clinical characteristics of participants who underwent cochlear implantation.

Measure		Number and percentage
**Gender**		
	Male	27 (51.90%)
	Female	25 (48.10%)
**Age (years)**		
	0–2 years	12 (23.10%)
	2–4 years	24 (46.20%)
	>4 years	16(30.80%)
**Category of auditory performance**		
	Grade 0	4 (7.70%) (Male: 2; Female: 2)
	Grade 1	12 (23.10%) (Male: 7; Female: 5)
	Grade 2	25 (53.80%) (Male: 13; Female: 12)
	Grade 3	11 (15.40%) (Male: 5; Female: 6)

Dataset 2 includes 25 females and 27 males. There were 12 subjects aged range of 0–2 years (23.10%), 24 subjects of 2–4 years (46.20%), and 16 of > 4 years (30.80%). The number of subjects in Grade 0, 1, 2, and 3 of CAP was 4 (7.70%), 12 (23.10%), 25 (53.80%), and 11 (15.40%), respectively.

### Optimal Model Parameters

Table 3 shows the optimal KPCA parameters and internal hyperparameters of each classifier. Sigmoid kernels are more suitable for SVM and KNN classifiers, and their optimal number of features is six and five, respectively. Linear kernels are more suitable for LR, and the optimal number of features is ten. For SVM classifier, the optimal hyperparameters are *C* = 0.62, gamma = 4.268, and Kernel = “RBF”. The optimal number of neighbors is nine in KNN. The optimal type of penalty is “L2”, and the solver is “L-BFGS” in LR.

**TABLE 3:** Optimal parameters of dimensionality reduction and internal hyperparameters of the classifiers.

Classifier	Parameters of PCA		Internal hyperparameters
	Kernel function	Feature number	
SVM	Sigmoid	6	C = 0.62, gamma = 4.268, Kernel = RBF
KNN	Sigmoid	5	n_neighbors = 9
LR	Linear	10	Penalty = ‘L2’, solver = ‘L-BFGS’

### Classification Performance of Healthy Controls vs. Sensorineural Hearing Loss

**FIGURE 3:**
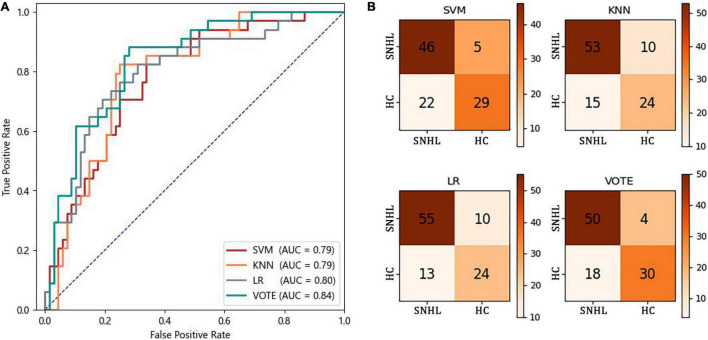
Classification performance of HC vs. SNHL. **(A)** ROC curves; **(B)** confusion matrix.

Figure 3 shows the receiver operating characteristic curve and the confusion matrix of the classification of HC vs. SNHL by three machine-learning methods and their voting. The AUCs of SVM, KNN, and LR are 0.79, 0.79, and 0.80, respectively. No significant difference was found among the three classifiers (DeLong test, *p* > 0.05). After the voting, the AUC increased to 0.84, higher than every single classifier, although significance is not observed (DeLong test, *p* > 0.05). In the confusion matrix of the voting method, 18 of 68 SNHL are wrongly predicted as HC and 4 of 34 HC are wrongly predicted as SNHL.

Table 4 shows the precision, recall, F1-score, accuracy, and AUC of three machine-learning methods and their voting. As expected, the voting yields higher performance compared to the three single machine-learning methods for all measures: precision of 0.81; recall of 0.77; F1-score of 0.78; and accuracy of 0.77.

**TABLE 4:** Classification performance of each classifier and voting.

Classifiers	Precision	Recall	F1-score	Accuracy	AUC
SVM	0.77	0.72	0.72	0.72	0.78
KNN	0.77	0.76	0.77	0.76	0.79
LR	0.70	0.72	0.70	0.72	0.80
VOTE	0.81	0.77	0.78	0.77	0.84

### Prediction Performance of Category of Auditory Performance After Cochlear Implantation

Table 5 lists the fitting information of CAP after CI by multiple logistic regression (MLR). The final value of MLR using –2 Log likelihood as the fitting condition reaches 54.716, and the interception is 126.504. For the likelihood ratio, the value of Chi-square, degree of freedom, and significance is 71.788, 42, and 0.003, respectively. The significance is 0.003, considerably below 0.05, indicating a high degree of fitting. For the pseudo R-squared test, the final value of Cox-Snell, Nagelkerke, and McFadden reaches 0.749, 0.821, and 0.567, respectively. All values are close to 1.0 and larger than 0.5, indicating a good fitting result.

**TABLE 5:** Fitting information of CAP after CI by multiple logistic regression.

Model	Model fitting conditions: -2 Log-likelihood	Likelihood ratio			Pseudo R-squared		
		Chi-square	Degree of freedom	Significance	Cox-Snell	Nagelkerke	McFadden
Final value	54.716 (Interception 126.504)	71.788	42	0.003	0.749	0.821	0.567

Defining *w*_*j*_⋅*x*_*i*_ as *G_j_*, the fitted parameters are given as follows.


(2) $$G_{0}=-233.816+75.413\,{\text{f}}_{1}+4.407\,{\text{f}}_{2}-16.785\,{\text{f}}_{3}+141.98\,{\text{f}}_{4}$$

$$-32.187\,{\text{f}}_{5}+42.805\,{\text{f}}_{6}+13.628\,{\text{f}}_{7}+47.64\,{\text{f}}_{8}+82.779\,{\text{f}}_{9}+61.62\,{\text{f}}_{10}$$

$$-3.428\,\mathrm{ABR}-1.525\,\mathrm{female}+37.286\,{\text{age}}_{1}+12.029\,{\text{age}}_{2}$$

(3) $$G_{1}=-10.13+6.551\,{\text{f}}_{1}+3.022\,{\text{f}}_{2}-0.636\,{\text{f}}_{3}-3.111\,{\text{f}}_{4}+2.961\,{\text{f}}_{5}$$

$$-3.749\,{\text{f}}_{6}-6.372\,{\text{f}}_{7}+5.079\,{\text{f}}_{8}-3.605\,{\text{f}}_{9}-3.283\,{\text{f}}_{10}$$

$$-13.44\,\mathrm{ABR}-1.193\,\mathrm{female}+1.422\,{\text{age}}_{1}-1.484\,{\text{age}}_{2}$$

(4) $$G_{2}=0.717-1.347\,{\text{f}}_{1}-2.464\,{\text{f}}_{2}+3.478\,{\text{f}}_{3}-0.66\,{\text{f}}_{4}-7.534\,{\text{f}}_{5}$$

$$-1.727\,{\text{f}}_{6}-6.82\,{\text{f}}_{7}+5.26\,{\text{f}}_{8}-3.663\,{\text{f}}_{9}+0.81\,{\text{f}}_{10}$$

$$+7.555\,\mathrm{ABR}-1.207\,\mathrm{female}+0.266\,{\text{age}}_{1}-0.169\,{\text{age}}_{2}$$

(5) $$G_{3}=0$$

Here, f_1_,f_2_,…,f_10_ are the features After dimensionality reduction. The value age_1_ represents 1 if it is in the range of 0–2 years; otherwise, it is 0. The value age_2_ represents 1 if it is in the range of 2–4 years; otherwise it is 0. If the subject is a female, the value female is 1; otherwise, it is 0.

*P_i_* represents the probability that the subject belongs to the *i*-th category of auditoria performance.


(6) $$P_{i}=\frac{\text{exp}\,\left(G_{i}\right)}{\sum_{j=0}^{3}\text{exp}\,\left(G_{j}\right)}$$

Using Eqs (2–6) and the input data off_1_,f_2_,…,f_10_, ABR, age_1_, age_2_, and female, we obtain the statistical results listed in Table 6. The prediction accuracy to CAP of 0, 1, 2, and 3 is 100.0, 83.3, 92.0, and 54.5%. For CAP of 0, 1, and 2, the prediction is very good. However, the prediction performance is poor for CAP of 3. 4 of 11, and 1 of 11 SNHL patients with CAP of 3 were wrongly predicted as CAP 2 and 1, respectively. This indicates that there is a minor difference between functional netWorks of CAP 2 and CAP 3. The average accuracy for the prediction of the four categories is 82.7%.

**TABLE 6:** Prediction of CAP after CI.

True\Prediction	0	1	2	3	Accuracy
0	4	0	0	0	100.0%
1	0	10	1	1	83.3%
2	0	1	23	1	92.0%
3	0	1	4	6	54.5%
Average	-	-	-	-	82.7%

## Discussion

The preoperative evaluation of children with SNHL about to undergo cochlear implantation was performed using rs-fMRI images, including auxiliary diagnosis and prediction of postoperative recovery. By combining functional connections measured by fMRI and machine learning methods, the classification models were constructed, and the highest AUC of differentiating SNHL from HC reached 0.84. Furthermore, the multiple logistic regression model was obtained, which predicts CAP after CI in SNHL with an average accuracy of 82.7%.

### Diagnosis of Sensorineural Hearing Loss by the Combination of Functional Brain Network and Machine Learning

Numerous studies showed that brain connections and functions of SNHL patients undergo numerous changes compared to the control group; however, their conclusions are not consistent (Kral and O’Donoghue, 2010; Huang et al., 2015; Liu et al., 2015; Shi et al., 2016; Xu et al., 2016; Tobyne et al., 2017; Tarabichi et al., 2018). Our classification models differ from previous studies, as they differentiate SNHL from HC on account of all differences between the two groups, which are embedded in the principal components extracted from FCs in the brain. Specific changes of FCs and functions of brain regions cannot be traced back due to the nature of kernel PCA.

Due to the complexity of the etiology of congenital SNHL, the diagnosis and cause determination of congenital SNHL often require a comprehensive evaluation combined with multiple diagnostic methods. Imaging methods are mostly employed to detect anatomical structures of the cochlea, labyrinth, and cranial nerves (Mulders et al., 2015; Puschmann and Thiel, 2017; Vos et al., 2017; Xia et al., 2017; Tang et al., 2019). Anatomical abnormalities can help confirm the cause of SNHL. Furthermore, in some cases, these abnormalities may completely exclude surgical intervention.

However, previous studies have shown that in imaging examinations, the diagnostic rate of HRCT is estimated to be 30% (Chen et al., 2014), and the effect of MRI is slightly higher. Our study focuses on the identification of SNHL based on the characteristics of brain function connections. The classification accuracy can reach 0.77, which outperforms traditional imaging methods. This may help doctors diagnose SNHL early and make clinical decisions.

### Prediction of Category of Auditory Performance After Cochlear Implantation in Sensorineural Hearing Loss by Multiple Logistic Regression

Previous studies (Woolley et al., 1997; Jackson et al., 2015) showed that the decision whether to carry out surgical interventions generally relies on the discovery of abnormal anatomical structures, and there is no clear decision basis for the types of abnormalities. This study predicts the CAP as a prognostic measure after CI in SNHL, based on the characteristics of brain function connections. The average accuracy achieves 82.7%. This may contribute to decision-making in surgical intervention, and help establish psychological expectations for patients and their families and thus reduce unnecessary medical disputes.

The deprivation of auditory input in the early sensitive period has a significant impact on the internal organization of the brain, with lasting effects. The FCs we choose can represent changes in the patient’s brain function, and the age at the time of surgery is an important parameter for predicting postoperative effects. Simultaneously, to consider the different development speeds due to gender differences, gender is used as a reference factor (Sharma and Campbell, 2011; Kral and Sharma, 2012).

### Limitations and Future Work

This study has certain limitations. First, the prediction accuracy of this study is not particularly high, which may be related to the large number of younger subjects whose brain connections and structural changes are not yet evident. All children in this study are patients with severe congenital deafness, and the diagnostic effect for mild cases is temporarily unknown. Second, currently, it is not possible to determine the specific location of abnormal brain regions based on the diagnostic results, it is impossible to prompt the cause, and further research is required for validation. Third, the evaluation index CAP of the surgical effect generally contains eight levels, whereas the evaluation results obtained only range from 0 to 3, such that it is impossible to make more accurate regression predictions. This means that if there is a pathology with a postoperative effect that is better than grade 3, it cannot be predicted. Fourth, the participants ranged in age from 12 to 137 months, which is when the human brain undergoes dramatic changes. Narrowing the age range may make the present results more convincing while decreasing the number of participants.

In the future, research will be carried out in the direction of improving the diagnostic accuracy and disease traceability to provide an important basis for the diagnosis of the cause of SNHL. As the volume of data increases, we expect that the predictive ability of the postoperative effect will be enhanced, which may provide useful support for the diagnosis and treatment of SNHL.

## Conclusion

We used functional brain connections to identify sensorineural hearing loss and predict the outcome of cochlear implantation by machine learning methods. The constructed machine learning models, in particular the one after voting, accurately classified participants into SNHL and HC. The constructed multiple logistic regression model enables the prediction of CAP after CI in SNHL with a high average accuracy. These models might help improve the diagnosis of SNHL through fMRI images and prognosis prediction of CI in SNHL.

## Data Availability Statement

The datasets presented in this article are not readily available because they must be approved by the Ethics Committee of The Affiliated Hospital of Guizhou Medical University. Requests to access the datasets should be directed to HY, 331693861@qq.com.

## Ethics Statement

The studies involving human participants were reviewed and approved by The Affiliated Hospital of Guizhou Medical University. Written informed consent to participate in this study was provided by the participants’ legal guardian/next of kin.

## Author Contributions

QS performed experiments and analyzed the data along with SQ. SQ, WQ, HZ, and HY conceived the study, presented the results, and wrote the manuscript along with QS. YY, HZ, and HY collected and analyzed the data. LY and CJ supervised the algorithm development and analyzed the data. All authors read and approved the final manuscript.

## Conflict of Interest

The authors declare that the research was conducted in the absence of any commercial or financial relationships that could be construed as a potential conflict of interest.

## Publisher’s Note

All claims expressed in this article are solely those of the authors and do not necessarily represent those of their affiliated organizations, or those of the publisher, the editors and the reviewers. Any product that may be evaluated in this article, or claim that may be made by its manufacturer, is not guaranteed or endorsed by the publisher.

## Abbreviations

ABR: auditory brainstem response
AUC: area under curve
BOLD: blood-oxygen-level-dependent
CAP: category of auditory performance
CI: cochlear implantation
DPABI: data processing & analysis for brain imaging
FA: flip angle
FC: functional connection
fMRI: functional magnetic resonance imaging
FOV: field of view
HC: health control
HRCT: high-resolution computed tomography
KNN: k-nearest neighbor
KPCA: kernel principal component analysis
LR: logistic regression
MLR: multiple logistic regression
MRI: magnetic resonance imaging
PCA: principal component analysis
rs-fMRI: resting-state fMRI
SIR: speech intelligibility rate
SNHL: sensorineural hearing loss
SVM: support vector machine
TE: echo time
TR: repetition time.
